# Age- and Gender-Specific Differences in the Seasonal Distribution of Diabetes Mortality in Shandong, China: A Spatial Analysis

**DOI:** 10.3390/ijerph192417024

**Published:** 2022-12-18

**Authors:** Wenxiu Zheng, Jie Chu, Jie Ren, Jing Dong, Hilary Bambrick, Ning Wang, Kerrie Mengersen, Xiaolei Guo, Wenbiao Hu

**Affiliations:** 1 Ecosystem Change and Population Health Research Group, School of Public Health and Social Work, Queensland University of Technology, Brisbane, QLD 4059, Australia; 2 The Department for Chronic and Non-Communicable Disease Control and Prevention, Shandong Center for Disease Control and Prevention, Jinan 250014, China; 3 National Centre for Epidemiology and Population Health, Australian National University, Canberra, ACT 2601, Australia; 4 National Centre for Chronic and Noncommunicable Disease Control and Prevention, Chinese Centre for Disease Control and Prevention, Beijing 100050, China; 5 School of Mathematical Sciences, Queensland University of Technology, Brisbane, QLD 4059, Australia

**Keywords:** diabetes, Shandong, spatial, cluster, season

## Abstract

Diabetes mortality in Shandong is higher than the national average in China. This study first explored diabetes mortality variation spatially at the county/district level among adults aged over 30 years in terms of age and gender, specifically by season. Daily diabetes mortality data were collected from 31 mortality surveillance points across Shandong Province in 2014. A geographic information system, spatial kriging interpolation and a spatial clustering method were used to examine the spatial patterns of diabetes mortality at the county/district level by season. Sensitivity analysis was conducted using diabetes mortality data from 10 mortality surveillance points from 2011 to 2020. As a result, the total diabetes mortality in eastern counties/districts was the highest (relative risk (RR) of cluster: 1.58, *p* = 0.00) across the whole province. For subgroups, women had higher mortality (16.84/100,000) than men (12.15/100,000), people aged over 75 years were the most vulnerable (93.91/100,000) and the highest-risk season was winter. However, the mortality differences between winter and summer were smaller in eastern and coastal regions than in other regions for all gender- and age-specific groups. The findings provide further evidence for early warning and precision preventative strategies for diabetes mortality in different regions of Shandong Province. Future research is required to identify the risk factors for diabetes and understand the differences in the social and environmental contexts.

## 1. Introduction

The burden of diabetes is increasing worldwide, characterized by high levels of blood glucose, and poses a serious threat to global societies [1]. The updated 2030 estimate of people with diabetes is 552 million around the world (10% of the world’s adult population) [2]. With symptoms such as polyuria, polydipsia and fatigue, the long-term effects or late diagnosis of diabetes can induce complications that seriously decrease the quality of daily life, such as diabetes nephropathy, neuropathy, retinopathy, and other vascular complications [1,3]. From 2000 to 2019, global deaths due to diabetes increased by 3%, whereas deaths from chronic respiratory diseases, cardiovascular disease and cancer dropped by 37%, 27% and 16%, respectively [4]. For China, 1.7 million deaths were caused by diabetes in 2019, accounting for 11.15% of global diabetes deaths [5].

Shandong is the second-largest population province in China with a population of around 100 million [6]. Diabetes was ranked as the sixth-highest cause of death in the 2016 Report on Disease and Health Status of Residents in Shandong, with higher mortality than the national average [7]. In 2015, diabetes mortality was reported at 13.86/100,000 in Shandong, whereas the rate across China in 2016 was 10.30/100,000 [8,9]. Therefore, given the large number of vulnerable populations across Shandong, it would be of great interest to investigate the spatial and temporal pattern of the diabetes burden to provide suggestions for more targeted health management and promotion policies.

However, to date, previous studies only focused on the prevalence of diabetes through surveys in several cities in Shandong Province [8,10,11]. In the past ten years, only two studies explored diabetes mortality in Shandong, but they did not involve spatial analysis or consider age- or gender-specific diabetes mortality differences by season. One study in Jinan city found that diabetes mortality was 6.06/100,000, and the mortality in men was lower than in women (the ratio was 0.84) from 2011 to 2015, without spatial analysis [12]. Another study showed diabetes mortality across Shandong in 2015 at the city level with no subgroup or spatial pattern exploration [8]. Hence, little evidence could be found for Shandong Province regarding the spatial or seasonal distribution of diabetes mortality among genders and age groups in the last decade.

The key objective of this study is to evaluate the spatial pattern of diabetes mortality and its seasonal distribution among subgroups by age and gender to identify potential high-risk areas and periods. The findings could provide crucial information about current spatial and seasonal discrepancies and suggest future interventions.

## 2. Materials and Methods

### 2.1. Data Source

Diabetes deaths were extracted from the China National Death Surveillance System. The system covers 605 death surveillance points at the county/district level across China, after expanding from 161 points in 2013. The surveillance points were selected from 31 provinces by a multistage stratification based on the sociodemographic characteristics of the population [13]. The cause of death is registered by applying the rules in the tenth revision of the International Classification of Diseases (ICD-10) [14]. Trained and qualified doctors at all medical institutions in the surveillance counties/districts are responsible for reporting death cases of all ages to the Center for Disease Control and Prevention (CDC) through an internet-based system [15,16].

In order to reduce the potential bias caused by spatial estimation, we chose 2014 as the study period because there were 31 accessible surveillance data points, which is the maximum number of surveillance points from 2011 to 2020. In total, it is a population of 15,184,921 individuals aged over 30 years old. Diabetes death cases were diagnosed with diabetes being the underlying cause of death by qualified and trained medical staff and then were registered in the system by ICD codes: E10-E14 (diabetes mellitus, E10: type 1 diabetes mellitus; E11: type 2 diabetes mellitus; E12: malnutrition-related diabetes mellitus; E13: other specified diabetes mellitus; E14: unspecified diabetes mellitus) [13,14]. For populations specified by each surveillance point, age and gender were collected by Shandong CDC from the national registration system. Sensitivity analysis was performed using 2011–2020 data to check the robustness of the results.

### 2.2. Data Analysis

Due to few non-null values of diabetes cause of death under 30 years of age, this study only included death cases aged over 30 years old to identify seasonal and spatial patterns. ArcGIS (version 10.8) was used to map spatial patterns of diabetes mortality by age and gender. The Joinpoint Regression model (Joinpoint Regression Program, Version 4.9.1.0) was used to investigate trend changes in mortality by age groups. The significance of change was tested by a Monte Carlo permutation method [17]. The population aged over 30 years old was divided into three age groups, i.e., 30 to 60 years, 60 to 75 years and over 75 years. Seasons in this study were defined as spring (March to May), summer (June to August), autumn (September to November) and winter (December to February).

Ordinary kriging interpolation was used to depict the spatial variability in diabetes mortality [18]. The zonal function in ArcGIS converted estimated kriging data into the county/district level. Moran’s *I* was used to assess the overall spatial autocorrelation, and the Local Indicators of Spatial Association (LISA) map was used to detect areas of high or low mortality [19,20]. As a measure of spatial autocorrelation, Moran’s *I* can indicate the correlation between neighboring characteristics of a similar phenomenon [20]. Moran’s *I* value varies from −1 to 1, where 1 indicates a maximum positive association, −1 indicates a maximum negative association and zero means a completely random spatial pattern. Although Moran’s *I* identifies the type and strength of spatial autocorrelations, it cannot reveal the location of the significant spatial clusters and outliers. In this case, a LISA map was used through the Geoda software (subversion 1.20.0.20) to detect the spatial clusters by the high-high type (hot spot) and the low-low type (cold spot) and to detect the spatial outliers by the high-low type and the low-high type [19].

SaTScan (version 10.1) was used to identify spatial clusters. SaTScan has been widely used in spatial clustering analysis by conducting discrete scan statistics [21]. In the current study, purely spatial scanning analysis with both low-rate and high-rate areas was conducted with the discrete Poisson model for the population at risk. All findings were mapped in ArcGIS.

To further explore the characteristics of the spatial distribution, negative binomial regression was used to assess the potential associations between age- and gender-specific diabetes mortality and geographical information (i.e., longitude, latitude and elevation [22]) due to the overdispersion of the mortality data (ratio of residual deviance and degrees of freedom > 1). The analysis was conducted by using R software (version 4.2.1).

Finally, to confirm the robustness of the spatial and temporal results in this study, data from ten surveillance points in Shandong Province from 2011 to 2020 were selected to conduct similar spatial and seasonality exploration.

## 3. Results

### 3.1. Descriptive Analyses

**Figure 1:**
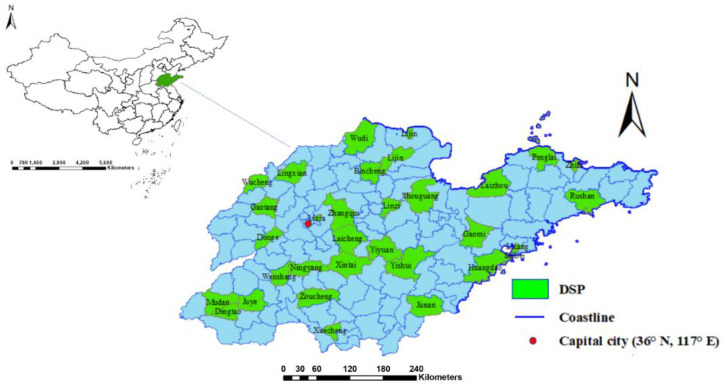
Surveillance points in Shandong Province, China, 2014.

**Figure 2:**
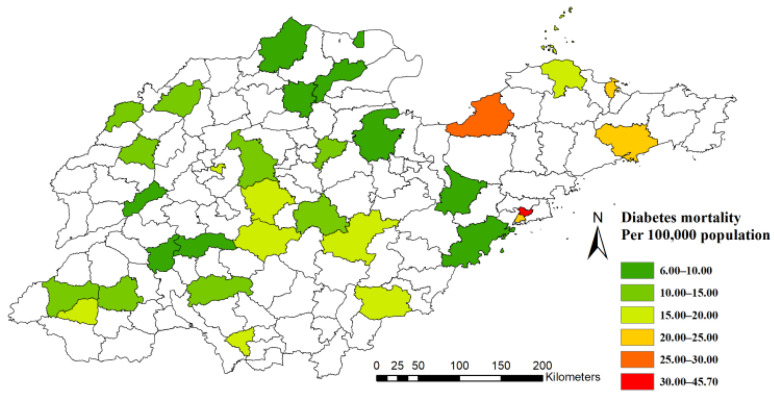
Diabetes mortality in 31 surveillance points in Shandong Province, China, 2014.

Figure 1 shows the distribution of 31 surveillance points at the county/district level in 2014 across Shandong Province. A total of 2201 cases (aged over 30 years old) were recorded as diabetes-caused deaths in the Death Surveillance System in Shandong Province in 2014. Figure 2 maps the distribution of mortality in 2014 at the county/district level. The mortality of diabetes varied in subpopulations and seasons (Table 1). First, the mortality in women (16.84/100,000) was higher than it was in men (12.15/100,000). Second, mortality increased with age. The highest mortality was 93.91/100,000 in the group aged over 75 years old, and the lowest was 3.19/100,000 in the subpopulation aged 30 to 60 years. Third, mortality was consistently highest in the winter season for each subpopulation in this study.

### 3.2. Spatial Autocorrelation and Interpolation

**Figure 3:**
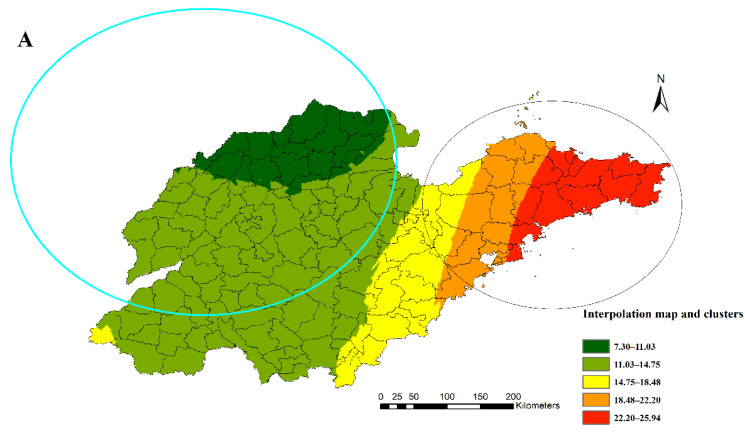

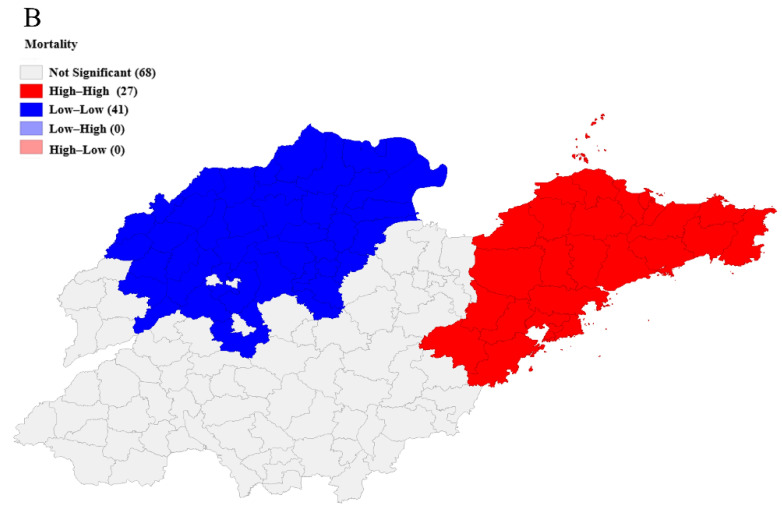
Kriging interpolation map of diabetes mortality (**A**) and Local Indicators of Spatial Association map (**B**) of all counties or districts in Shandong Province, China, 2014.

The result of spatial autocorrelation analysis shows that overall, there was a positive spatial autocorrelation (Local Moran’s *I* = 0.613, *p* = 0.02) between the mortality of diabetes among the 31 surveillance points. Then, the spatial interpolation of the yearly estimated rates was mapped with the statistically significant clusters from the SaTScan results; detailed cluster characteristics in this study can be found in Table S1. Figure 3A indicates the high-risk cluster (relative risk (RR): 1.58, including 27 counties) in the eastern region and the low-risk cluster (RR: 0.71, including 68 counties) in the north-western region of Shandong Province. The LISA map shows a similar spatial autocorrelation result (Figure 3B).

**Figure 4:**
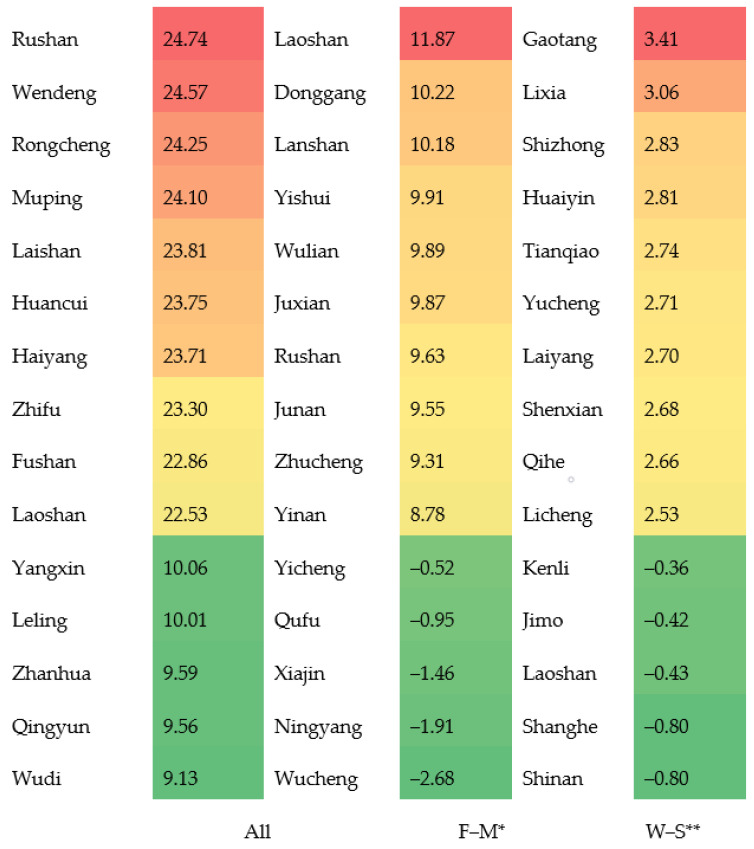
Rank of counties by estimated mortality of diabetes (top 10 and lowest 5) in Shandong Province, China, 2014; F−M*: rates in women minus rates in men; W−S**: difference between mortality in winter and mortality in summer (calculated as the mortality in winter minus mortality in summer).

Figure 4 lists the rank of counties with the highest ten and lowest five mortalities of diabetes for total, gender- and season-specific groups. Counties/districts in coastal and eastern cities, such as Weihai, Yantai and Qingdao, had the highest annual mortalities. Moreover, mortalities among women were much higher than among men in this region. Regarding the mortality difference between winter and summer, mortalities in eastern and coastal districts were ranked the lowest. Conversely, in western counties/districts, annual diabetes mortalities were relatively lower and the gender differentiations were less than those in the eastern counties/districts. However, the difference in mortalities between winter and summer was greater in western counties/districts compared with the difference in eastern counties/districts.

### 3.3. Spatial Clusters

**Figure 5:**
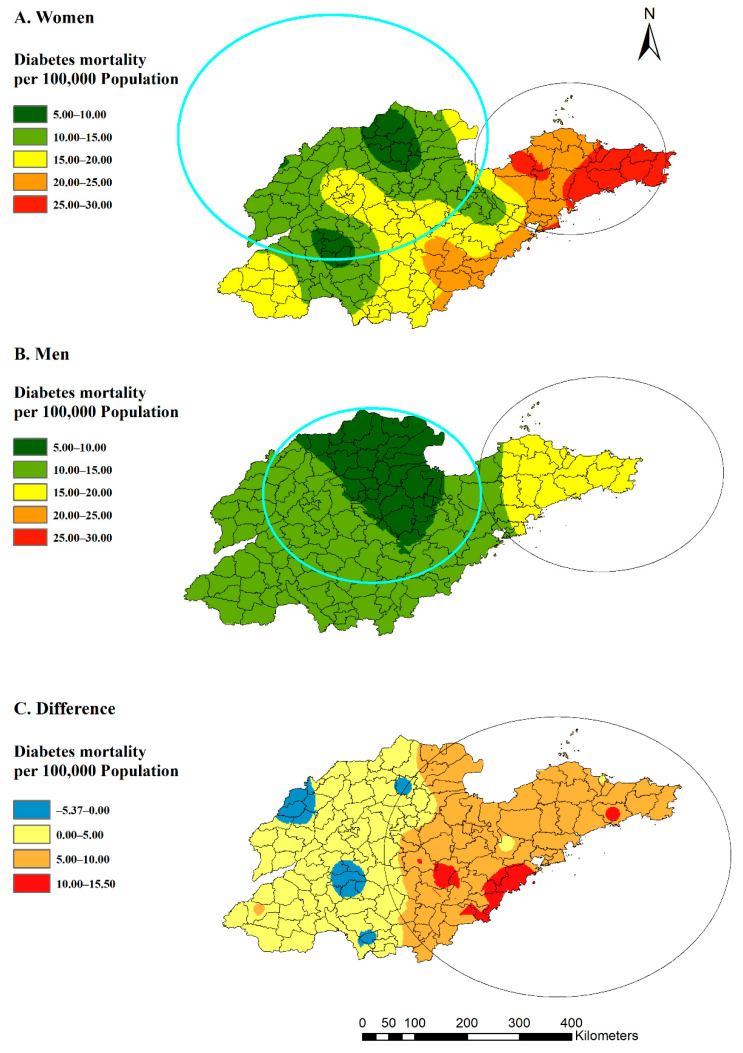
Estimated diabetes mortality by gender (**A**,**B**) and distribution of rates differences between women and men (**C**) with clusters in Shandong Province, China, 2014.

For spatial clusters by gender (Figure 5), the eastern region was a statistically significant high-risk region for both women (RR: 1.79, *p* = 0.00) and men (RR: 1.62, *p* = 0.00), and the northern region was included in the low-rate clusters for men (RR: 0.77, *p* = 0.00) and women (RR: 0.64, *p* = 0.00). In addition, the spatial clustering of the difference between women and men (mortality rates in women minus rates in men) in the whole year revealed that women could even be at lower risk than men in several western counties, which was significantly different from the eastern region (RR: 2.81, *p* = 0.00).

**Figure 6:**
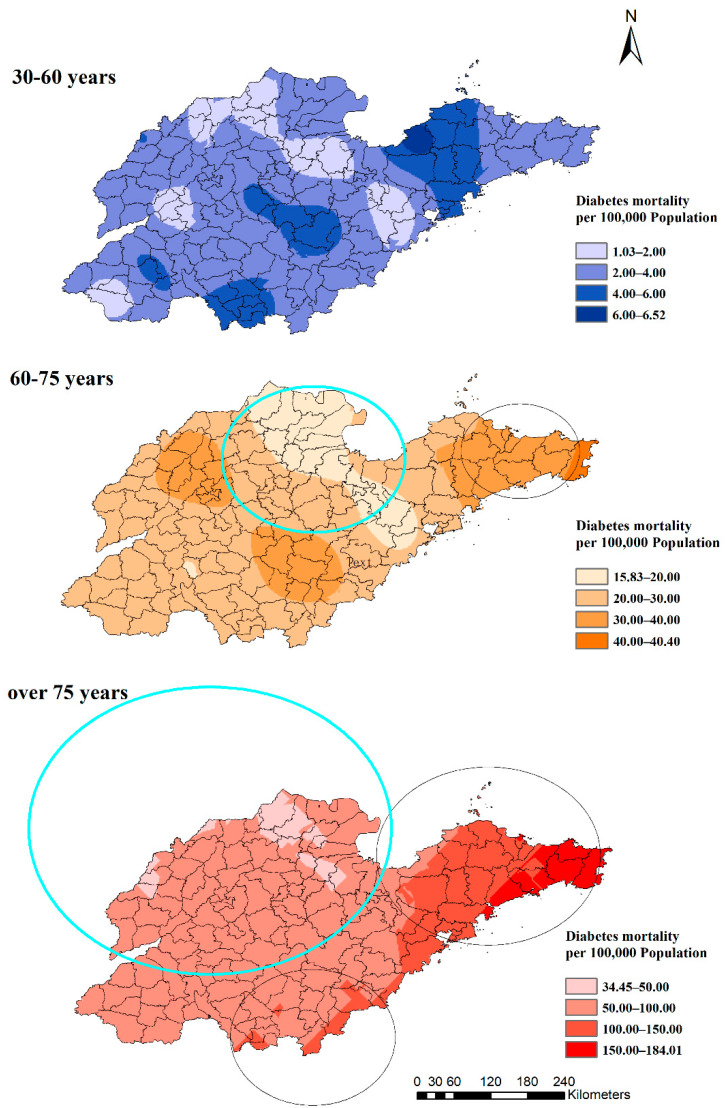
Estimated diabetes mortality by age groups with clusters in Shandong Province, China, 2014.

The clustering of diabetes mortality increased significantly with age. Among the three age groups, there was no cluster with the highest rate of 6.52/100,000 among the 30 to 60 years group. However, in the 60–75-year-old group, there were two statistically significant clusters, and the lowest rate was 15.83/100,000 (Figure 6). The high-rate cluster was in the eastern region (RR: 1.35, *p* = 0.00) and the low-rate cluster was in the northern region (RR: 0.78, *p* = 0.00). The group aged over 75 years had the biggest low- and high-rate clusters. The low-rate cluster included 67 counties (RR: 0.65, *p* = 0.00), of which, 33 counties were in the 60– 75-year-old group. Furthermore, two high-rate clusters included 16 and 24 counties (RR: 1.12, *p* = 0.04 and RR: 1.72, *p* = 0.00) separately in the over-75-year-old group, of which, 11 (RR:1.35, *p* = 0.00) were in the 60–75-year-old group.

### 3.4. Geographical Associations

From the results above, diabetes mortality shows a tendency to increase from west to east in Shandong Province. The associations between mortalities and geographical factors (longitude, latitude and elevation) were assessed by univariate and multivariate negative binomial regression (Table 2). Longitude was positively associated with mortalities (ORs > 1), which is consistent with the tendency shown in the maps above and indicates again that the eastern region of Shandong with higher longitudes could be a high-risk region. Elevation was evaluated to have a positive association with mortality in some groups, but was not statistically significant (*p* ≥ 0.05) in men and in the population aged 30–60 and over 75 years old. Latitude did not show significant associations with mortalities in this study.

### 3.5. Seasonality Pattern in Subgroups

**Figure 7:**
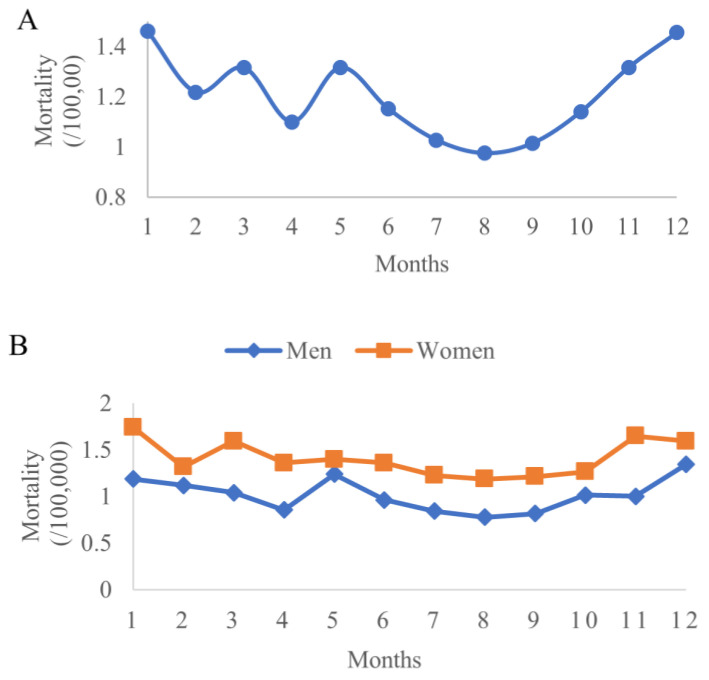
Average diabetes mortality rate in 31 surveillance points by months: total (**A**), by gender (**B**).

As shown in Figure 7, the data displayed a seasonal pattern of the average mortality in 31 points with the lowest in summer and the highest in winter. The rates by gender also showed the same patterns.

**Figure 8:**
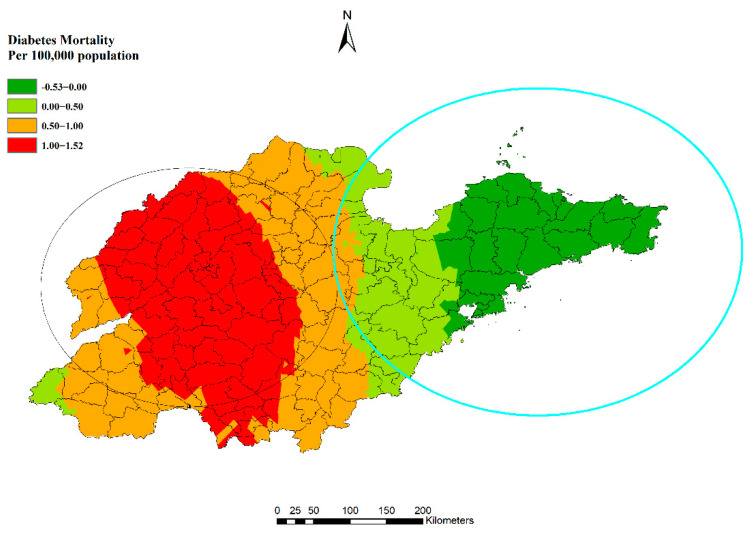
Differences in diabetes mortality between winter and summer in men.

However, regarding the difference between summer and winter at the county/district level, the patterns were different in the absolute rates. Although the eastern region remained as a high-rate area for both genders in winter (Figure S1), the western region experienced an increase from summer to winter (Figure S2). There were even lower mortalities in winter than in summer in some eastern counties for men (Figure 8).

**Figure 9:**
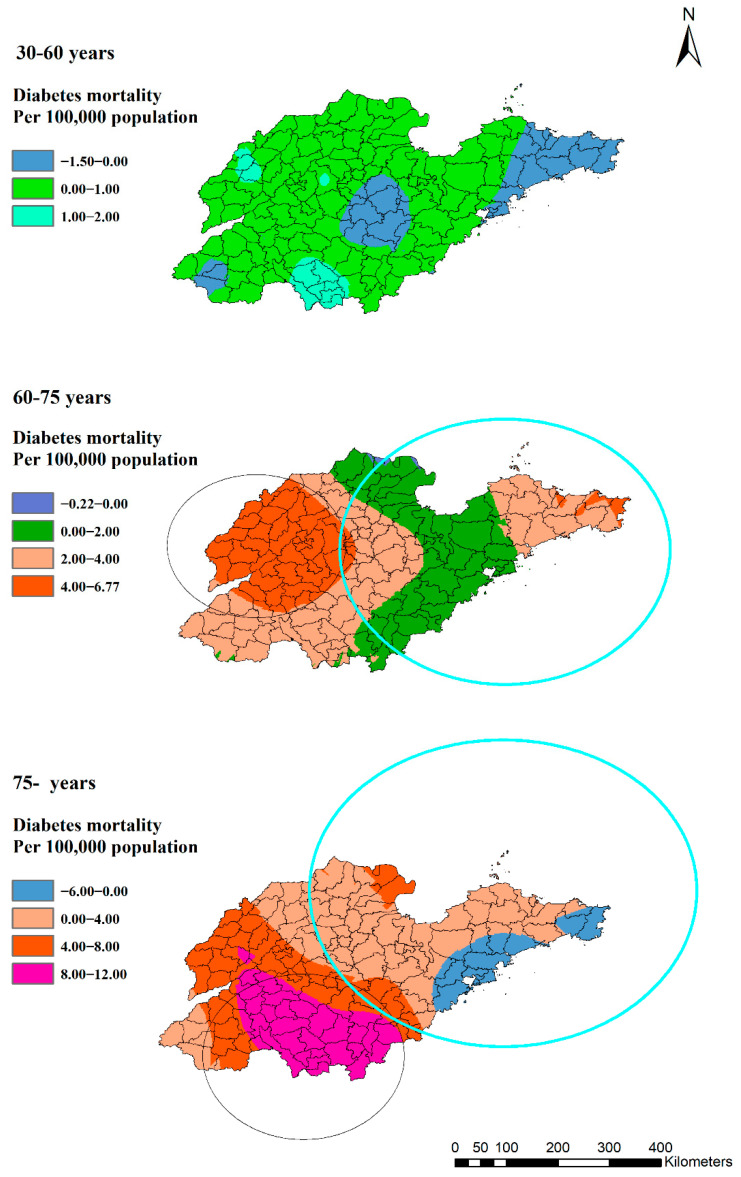
Differences in estimated diabetes mortality between winter and summer by age group with clusters.

A similar pattern was shown in different age groups (Figure 9). Eastern and coastal counties/districts experienced less difference between winter and summer than other parts. Moreover, the mortality in summer was higher than in winter in some counties in the east. Hence, the group aged 60 to 75 years old in the western region and the group over 75 years old in the southern counties/districts were high-risk populations in winter, even though these regions had lower mortalities throughout the year.

### 3.6. Sensitivity Analyses

In order to confirm the robustness of the spatial pattern results from 2014 in this study, mortality data from ten counties/districts from 2011 to 2020 were selected for sensitivity analysis. The results also showed the same seasonality pattern with winter having the highest rate of diabetes death among the population aged over 30 years (Figure S3). The eastern and coastal counties/districts, such as Shibei, Penglai and Rushan, also had the highest average mortality from 2011 to 2020 (Figure S4). Therefore, the spatial and seasonal patterns of average diabetes mortality in 10 points from 2011 to 2020 were similar to those in 31 points in 2014.

## 4. Discussion

In this study, women and people aged over 75 years were the most vulnerable subgroups to diabetes death in Shandong Province, especially in the eastern counties/districts. Western and southern counties/districts experienced a surge in diabetes mortality in winter for age-, gender-specific and overall population groups, whereas the seasonality pattern was not significant in eastern counties/districts.

The overall diabetes mortality rate in the 31 surveillance points was 14.19/100,000 in 2014 in Shandong Province. In the same year, age-standardized diabetes mortality in China was estimated to be 9.53/100,000 by the Global Burden of Disease (GBD) [5]. This confirms the higher diabetes burden in Shandong Province across the whole country.

One published piece of research on high-risk areas for diabetes mortality in Shandong in 2000 also revealed that the eastern region had a higher diabetes mortality rate; however, the conclusion for the western region was inconsistent with our findings [23]. This earlier study found both the western and northern parts had higher diabetes mortalities, which may indicate the change in diabetes mortality in these two regions from 2000 to 2014. For the eastern region, investigations in 2000 and 2014 confirmed it was a higher-rate region. Univariate regression results in the current study indicated that diabetes mortality increased with longitude, from west to east. According to the Shandong Statistical Yearbook, the gross domestic product per capita in coastal cities, such as Qingdao (96,524 CNY), Yantai (85,795 CNY) and Weihai (99,392 CNY), was higher than that in western cities, such as Liaocheng (42,482 CNY), Dezhou (45,641 CNY) and Binzhou (59,567 CNY) [24]. Therefore, the spatial trend was consistent with findings in previous studies which found that higher socioeconomic status groups had higher diabetes rates [25,26]. This may result from more diagnoses in the wealthy group with better access to medical care, and unhealthier lifestyles such as less occupational physical activity than that in the lower-income group [27]. In addition, the associations with elevation may indicate that the effects of cold temperatures could increase the risk of diabetes death, as temperatures decrease with altitude. The inference coincides with the finding that winter was the highest risk season in this study, but it is not consistent with a previous study of temperatures applied in Chongqing and Harbin, which concluded that diabetes mortality was more likely to occur in hot weather than cold [28].

This study found that mortality in women was higher than in men. However, from the GBD report, the mortality of males (10.8/100,000) was calculated to be higher than for females (8.97/100,000) in China in 2014 [5], which is contrary to the findings of the current study in Shandong Province. The result from a national study across China also shows that the age-standardized mortality rate for males (14.51/100,000) was higher than for females (12.73/100,000) in 2020 [29]. Shandong may have a different gender pattern of diabetes burden than nationally. Two possible explanations for the latter are that men may engage in more frequent exercise, which may decrease the risk of diabetes [30], and women in Shandong may have a longer life expectancy than nationally. According to previous reports, the life expectancy of Chinese women was 79.9 and 73.2 for men, whereas the data indicated 81.38 and 76.07, respectively, in Shandong Province [7,31]. Since diabetes mortality increases with age, peaking at ages over 75 years, the longer life expectancy of Shandong women may lead to the higher mortality of diabetes in the current study. Regarding age groups, diabetes mortality in the over 75 years old group was 3.56 times that of the 60–75-year-old group, confirming that the elderly group is most vulnerable to death from diabetes. A similar age distribution of diabetes deaths was also found in a cohort study in China from 1990–2017 based on the Global Burden of Disease 2017 Study and the national study by China CDC, indicating that diabetes mortality significantly increased with age [29,32]. Most people were aged over 65 at diagnosis, and the duration of diabetes could increase the risk of mortality [33]. Studies have shown that an insulin secretory defect is commonly observed due to interactive associations with age-related factors, such as impaired beta-cell compensation, adiposity, sarcopenia and decreased physical inactivity, which increases the risk of diabetes among elderly people [34,35,36]. Therefore, the prevention and control of diabetes in the elderly group can be an effective way to reduce the burden.

This seasonality analysis showed that winter was the surge period of diabetes death, especially in western and southern counties/districts. Previous studies of extreme temperatures demonstrated that cold weather in winter could increase the risk of diabetes (RR: 1.13, 95%CI: 1.03–1.34) [28]. However, as mentioned earlier, other studies have explored the influence of hot weather in summer and also found significant associations with increased diabetes risk [28,37,38,39,40]. In this study, mortality in the eastern coastal counties/districts did not change as significantly as in the western and the southern counties/districts. This may be due to the slighter temperature variability over seasons in the coastal region. For example, as calculated in this study [41], the temperature differences (between the mean temperature of summer and winter) were both around 25.00 °C in two inland cities, Binzhou and Dezhou, and around 22.50 °C in two coastal cities, Qingdao and Weihai.

The findings in the current study suggested more targeted and precise strategies for the future allocation of medical resources in Shandong Province. For example, clinicians and public health physicians should strengthen the prevention and treatment of diabetes among women and elderly people in Shandong. For the eastern counties/districts, it is necessary to enhance the prevention and management of diabetes due to the highest mortalities across the province. Moreover, for the diabetes patients living in the western and southern counties/districts, the surge in diabetes mortality in winter calls for raising public awareness of temperature variability at low temperatures and strengthening the prevention, mitigation and preparedness of diabetes.

To our knowledge, this is the first study that evaluated the spatial variation in diabetes mortality by season in Shandong Province using data that covered a population in death surveillance points with more than 15 million at the county/district level in the last ten years. However, the study has some limitations. First, we used surveillance data from 31 counties to interpolate the pattern of the whole province, which may lead to some differences from the real data, although sensitivity analysis yielded similar results to the main analysis. Second, this study only included the year 2014 as it encompassed the most surveillance points in the last ten years. With the development of the surveillance system in China, only ten counties/districts were constant in the system in Shandong from 2011 to 2020, which was less representative than the 31 points used in this study. Longer-term research needs to be conducted by processing the data from inconsistent points each year.

Further studies are recommended to assess the associations between these spatial and seasonal patterns in diabetes mortality and potential socioeconomic or environmental risk factors, such as temperature, green space, elevation and lifestyles, to explore the potential reasons for the different patterns in Shandong and to contribute to the future reduction in diabetes mortality. Bayesian spatiotemporal methods simultaneously considering the spatial and time correlation of the data are highly recommended to produce more comprehensive information for regional high-risk factors and give evidence to generate an early warning of diabetes mortality increase [42]. In addition, mobile health (mHealth) applications with artificial intelligence chatbots have been called for chronic disease surveillance and management with the increasing popularity of social media in daily life [43,44]. Studies indicate that applying mHealth can track blood glucose and elicit beneficial behavior to enhance the control of diabetes for individuals [45,46]. With the further support of high-risk environmental early warning information, mHealth applications have more promising potential to decrease the future burden of diabetes.

## 5. Conclusions

This study suggested that the spatial and seasonal patterns in diabetes mortality varied among age- and gender-specific subgroups across Shandong Province. Potential associations of social and environmental factors with spatial and temporal patterns should be explored in further studies. The findings provide important information for future regional diabetes medical policy modification and resource allocation.

## Figures and Tables

**Table 1:** The distribution of diabetes death by gender, age and season in 31 death surveillance points in Shandong Province, China, 2014.

Characteristics	No. of Death (*n*, %)	Population	Rate (per 100,000)
Gender			
Men	923	7,595,650	12.15
Spring	237 (26%)		3.12
Summer	195 (21%)		2.57
Autumn	215 (23%)		2.83
Winter	276 (30%)		3.63
Women	1278	7,589,271	16.84
Spring	330 (26%)		4.35
Summer	284 (22%)		3.74
Autumn	312 (24%)		4.11
Winter	352 (28%)		4.64
Age			
30–60	351	11,001,247	3.19
Spring	94 (27%)		0.85
Summer	78 (22%)		0.71
Autumn	72 (21%)		0.65
Winter	107 (30%)		0.97
60–75	812	3,078,369	26.38
Spring	217 (27%)		7.05
Summer	162 (20%)		5.26
Autumn	191 (24%)		6.20
Winter	242 (30%)		7.86
75–	1038	1,105,305	93.91
Spring	256 (25%)		23.16
Summer	239 (23%)		21.62
Autumn	264 (25%)		23.88
Winter	279 (27%)		25.24
Total (30–)	2201	15,184,921	14.49

**Table 2:** The associations between diabetes mortality with longitude, latitude and elevation (100 m) in 31 surveillance points, crude and adjusted odds ratio (OR) and 95% confidence interval (CI) in the negative binomial model (longitude, latitude and elevation (100 m)).

Variables	Crude OR (95%CI)						Adjusted OR (95%CI)					
	All	Gender		Age			All	Gender		Age		
		Men	Women	30–60	60–75	75–		Men	Women	30–60	60–75	75–
Longitude	1.13 (1.10–1.16)	1.12 (1.08–1.16)	1.18 (1.15–1.21)	1.18 (1.15–1.21)	1.03 (1.01–1.05)	1.14 (1.11–1.16)	1.11 (1.01–1.23)	1.08 (0.99–1.19)	1.17 (1.09–1.25)	1.04 (0.91–1.19)	1.01 (0.94–1.07)	1.21 (1.11–1.33)
Latitude	1.00 (0.95–1.06)	1.04 (0.97–1.11)	1.02 (0.96–1.09)	0.93 (0.83–1.04)	1.02 (0.98–1.06)	0.96 (0.91–1.02)	0.79 (0.64–0.96)	0.88 (0.73–1.06)	0.85 (0.73–0.98)	0.96 (0.73–1.26)	1.02 (0.89–1.17)	0.72 (0.59–0.87)
Elevation (100 m)	1.06 (1.01–1.13)	1.03 (0.98–1.08)	1.06 (1.01–1.11)	1.06 (0.99–1.13)	1.04 (1.00–1.0)	1.06 (0.99–1.13)	1.04 (0.98–1.10)	1.01 (0.96–1.06)	1.02 (0.98–1.07)	1.05 (0.98–1.13)	1.04 (0.99–1.08)	1.02 (0.97–1.08)

## Data Availability

Data are only available on reasonable request and approved by the Centre for Chronic and Non-communicable Disease Control and Prevention, Shandong Center for Disease Control and Prevention.

## Supplementary Materials

The following supporting information can be downloaded at: https://www.mdpi.com/article/10.3390/ijerph192417024/s1, Table S1: Characteristics of spatial clusters in this study; Figure S1: Mortality of men (A) and women (B) in winter in Shandong Province, China, 2014; Figure S2: Mortality difference between winter and summer in men (A) and women (B) groups respectively in Shandong Province, China, 2014; Figure S3: Average monthly mortality of men and women in Shandong province from 2011 to 2020; Figure S4: Diabetes mortality in ten surveillance points during 2011 to 2020 in Shandong Province.
