# Supplementary material for: Age- and Gender-Specific Differences in the Seasonal Distribution of Diabetes Mortality in Shandong, China: A Spatial Analysis
Source: Int J Environ Res Public Health. 2022 Dec 18;19(24):17024. doi: 10.3390/ijerph192417024 (PMC9779441; doi:10.3390/ijerph192417024)

Supplementary Materials:

Table S1. Characteristics of spatial clusters in this study

Total								
CLUSTER	LOC_ID	LATITUDE	LONGITUDE	RADIUS	NUMBER_LOC	P_VALUE	RR	POPULATION
1	370687	36.8587	121.103	156.432861	27	1.721E-14	1.579970545	2800000
2	371402	37.4417	116.378	230.52712	68	4.88057E-10	0.71059216	6800000
Men								
CLUSTER	LOC_ID	LATITUDE	LONGITUDE	RADIUS	NUMBER_LOC	P_VALUE	RR	POPULATION
1	370686	37.309	120.892	145.244446	24	1.05069E-07	1.623048327	2500000
2	370124	37.3219	117.2	195.219446	67	0.014	0.765870017	6700000
Women								
CLUSTER	LOC_ID	LATITUDE	LONGITUDE	RADIUS	NUMBER_LOC	P_VALUE	RR	POPULATION
1	370686	37.309	120.892	145.244446	24	1E-17	1.7881643836	2500000
2	371428	37.237	116.084	227.62974	67	1.22957E-12	0.6422959200	6700000
*Women-men								
CLUSTER	LOC_ID	LATITUDE	LONGITUDE	RADIUS	NUMBER_LOC	P_VALUE	RR	POPULATION
1	370211	36.1991	120.575	267.517344	66	1E-17	2.813391145	6700000
60-75								
CLUSTER	LOC_ID	LATITUDE	LONGITUDE	RADIUS	NUMBER_LOC	P_VALUE	RR	POPULATION
1	370522	37.1454	118.524	118.959879	33	6.15841E-06	0.7803030303	3300000
2	370611	37.2653	121.558	77.0645148	11	8.25898E-05	1.3535573532	1200000
75-								
CLUSTER	LOC_ID	LATITUDE	LONGITUDE	RADIUS	NUMBER_LOC	P_VALUE	RR	POPULATION
1	370686	37.309	120.892	145.244446	24	1E-17	1.72258019	2500000
2	371422	37.6801	116.806	234.063573	67	1E-17	0.651181016	6700000
3	371322	34.6485	118.315	110.68727	16	0.039	1.122627737	1600000

Table S1. (continued)

**W-S_men								
CLUSTER	LOC_ID	LATITUDE	LONGITUDE	RADIUS	NUMBER_LOC	P_VALUE	RR	POPULATION
1	371003	37.1522	122.001	186.024148	21	4.33136E-07	0.063580591	2200000
2	371726	35.5524	115.536	246.558443	65	0.001056712	2.245299145	6500000
W-S_60-75								
CLUSTER	LOC_ID	LATITUDE	LONGITUDE	RADIUS	NUMBER_LOC	P_VALUE	RR	POPULATION
1	371522	36.5815	116.171	139.080874	42	1.43649E-10	2.020966596	4200000
2	370214	36.4845	120.513	257.844536	64	6.58599E-06	0.575587287	6500000
W-S_75								
CLUSTER	LOC_ID	LATITUDE	LONGITUDE	RADIUS	NUMBER_LOC	P_VALUE	RR	POPULATION
1	370613	37.6558	120.836	297.654024	62	1E-17	0.361697078	6300000
2	370402	34.7996	117.347	160.609842	38	1E-17	2.535236396	3800000

*Women-men: mortality difference between women and men

**W-S: mortality difference between winter and summer

Figure S1. Diabetes mortality of men (A) and women (B) in winter in Shandong province, China, 2014

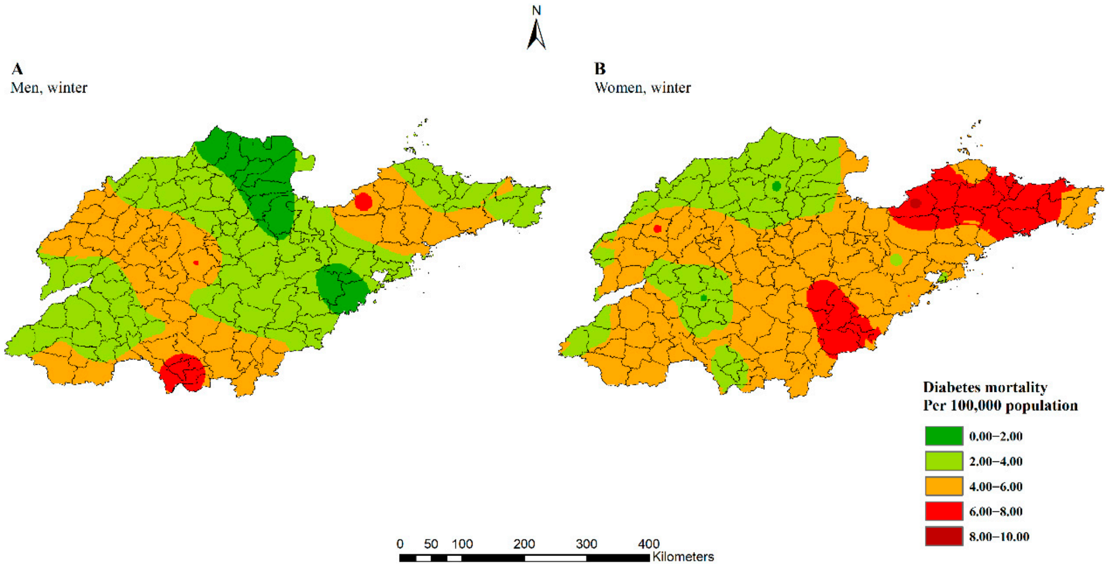

Figure S2. Diabetes mortality difference between winter and summer in men (A) and women (B) groups respectively in Shandong province, China, 2014

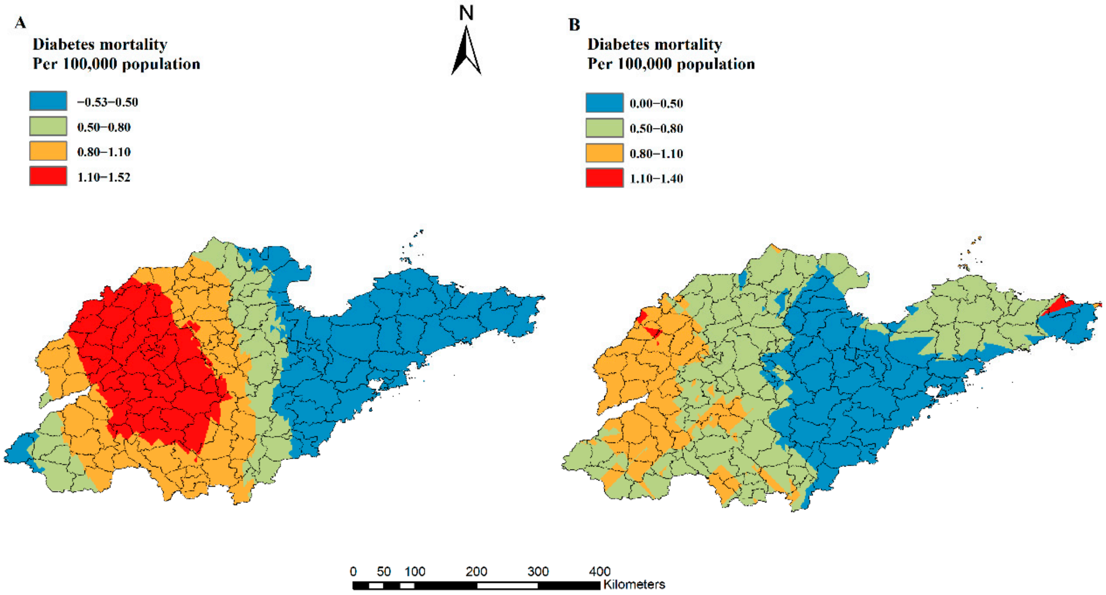

Figure S3. Average monthly diabetes mortality of men and women in Shandong province during 2011 to 2020

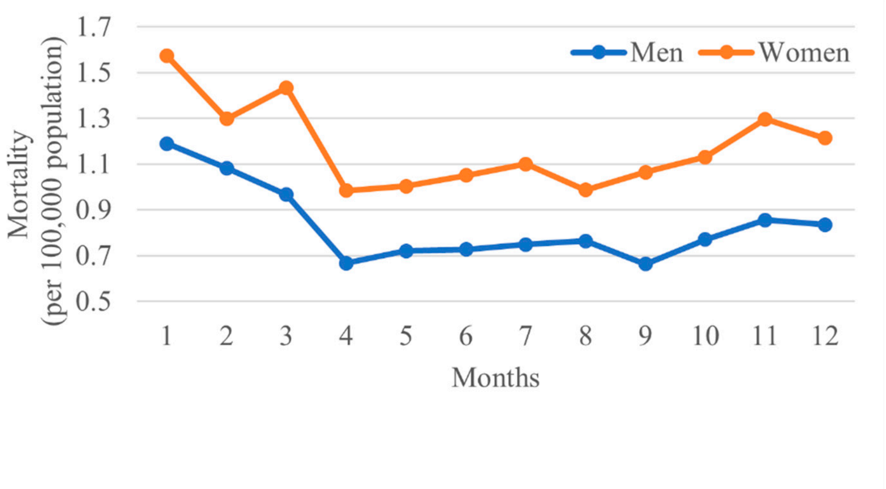

Figure S4. Diabetes mortality in ten surveillance points during 2011 to 2020, in Shandong province

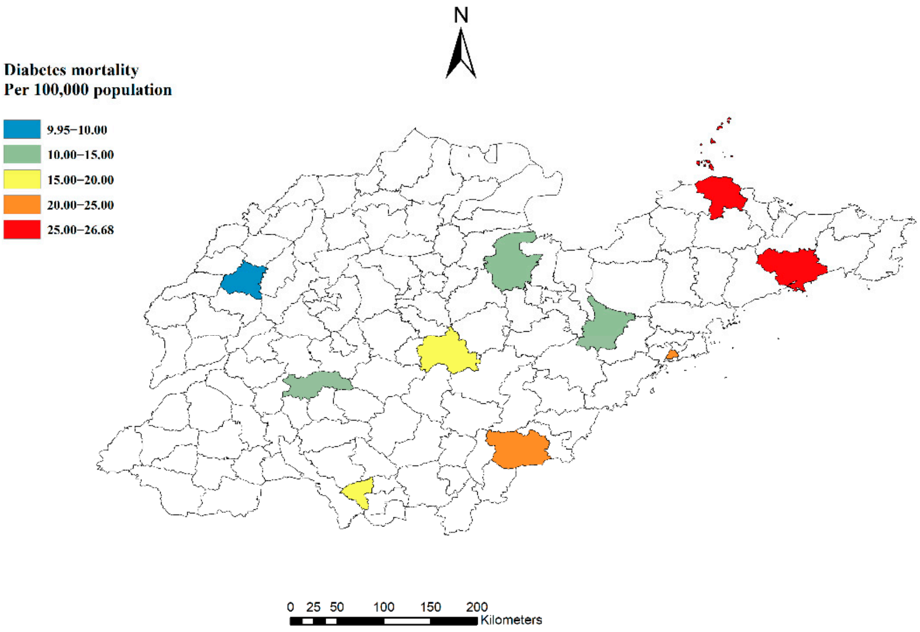

Supplement: Supplementary file 1 [file ijerph-19-17024-s001.zip › ijerph-2105291-supplementary.pdf]
